# Cost Analysis and Clinical Outcomes of Ambulatory Care Monitoring in Medicare Patients: Describing the Diagnostic Odyssey

**DOI:** 10.36469/9897

**Published:** 2015-02-11

**Authors:** Renée JG Arnold, Andrew Layton

**Affiliations:** 1 Quorum Consulting, Inc., New York, NY, USA; Mount Sinai School of Medicine, New York, NY; University of Colorado, Anschutz Medical Campus, Aurora, CO, USA; 2 Quorum Consulting, Inc., New York, NY, USA

**Keywords:** sequelae, monitoring, costs, holter, medicare, diagnosis, arrhythmia

## Abstract

**Objectives:** The diagnostic sequence and costs for arrhythmia detection utilizing Holter ambulatory ECG monitoring have not been well studied. The objective of the current study was to characterize the number of patients and associated costs incurred in the diagnosis, additional monitoring, clinical events and sequelae after an initial Holter monitor in Medicare patients with arrhythmia—the diagnostic odyssey.

**Methods:** We performed a retrospective, longitudinal claims analysis using a 5% random sample of Medicare beneficiaries’ claims from the Fee-for-Service (FFS) Standard Analytic Files. The analysis was limited to patients with full benefits for 1 year prior and 2 years post the index 24- or 48-hour Holter event, no prior arrhythmia or Holter.

**Results:** The group of greatest interest was the “No results” category, since these 1,976 patients (11.1% of the total 17,887 patients evaluated) reflected the failure of repeat Holter monitoring to either detect clinical events or diagnose disease. In spite of this failure, there was a total allowed charge of more than $45 million or slightly more than $23,000 per involved patient. When extrapolated over the entire Medicare FFS population, this category was estimated to cost more than $900 million over the 2-year study period.

**Conclusions:** Additional diagnostic paradigms need to be explored to improve upon these patient and system outcomes, where repeat monitoring frequently did not yield a diagnosis and patients continued to experience clinical events.

## BACKGROUND

Arrhythmias, such as atrial fibrillation (AF), are often asymptomatic, yet are associated with critical adverse outcomes, such as stroke. Moreover, survivors of a stroke with AF have a high risk of recurrent stroke.[1] Their management is expensive, with one source citing a cost of approximately $26 billion associated with AF.[2] Additionally, 25% of strokes are of unknown etiology and subclinical AF is often suspected to be the cause under these circumstances.[3,4] AF has been reported to be the most common type of arrhythmia and the incidence and prevalence of AF are exhibiting a continuing upward trend.[5]

If an arrhythmia is suspected, Holter Monitoring may be ordered. The Holter Monitor was first introduced into clinical practice in the 1960's and is a type of ambulatory electrocardiogram (AECG).[2,6-8] It is used to monitor ECG tracing continuously for a period of 24- to 48- hours after a patient experiences an arrhythmia that is not observed during an in-office ECG.[3,5] These types of events are often categorized as subclinical or asymptomatic.

There are two categories of Holter monitoring: 1) continuous recording of the ECG during the entire testing period and 2) event (loop) recording of the ECG only when the patient experiences symptoms of a potential arrhythmia. Although there does not appear to be any literature on patient satisfaction with Holter testing, the monitoring is largely non-invasive and the test is painless.[3] Despite the popularity of this test, the diagnostic yield (i.e. the detection of arrhythmias) may be low, varying from 1-22% in various case series.[8-11] Indeed, the common belief (although not well documented) is that repeat cardiac monitoring is frequent. Overall, the diagnostic sequence and its associated costs for arrhythmia detection utilizing Holter ambulatory ECG monitoring have not been studied to any extent. It is our goal here to characterize the diagnosis, additional monitoring, clinical events and sequelae of this detection system for arrhythmias. Importantly, we will translate these into costs that occur after an initial Holter monitor with a particular focus on Medicare patients.

## METHODS

### Objectives

Because of the dearth of specific information on the clinical use of Holter monitoring, the consequences of the use of this diagnostic tool, and, particularly, the costs associated with these activities and outcomes, we performed a large sample retrospective evaluation from a Medicare Fee-for-Service (FFS) database in order to evaluate these issues.

### Patients

We executed a retrospective, longitudinal claims analysis limited to 24- or 48- hour Holter patients with no prior arrhythmia and who underwent a "new" Holter monitor. Patients were followed over time to identify related diagnoses, additional monitoring events and related clinical events. The study period was defined for each patient as the two-year window from the first Holter event reported in 2009. Two years is a relevant timeframe to identify relevant follow-up events; a shorter timeframe may undercount such events, while a longer study period may identify fewer relevant events. Additionally, all monitoring codes were used to capture follow-up monitoring events, including global and technical components, hook-up and interpretation. Repeat monitor events (by American Medical Association [AMA] Current Procedural Terminology [CPT] code and date of service) must have been billed more than seven days apart from another monitoring event of the same type to limit the risk of double counting a single event.

To obtain the requisite data for our study, we used Medicare Standard Analytic Files (SAFs) provided by the Centers for Medicare and Medicaid Services. The SAFs contain a 5% random sample of beneficiaries' claims, which includes United States (U.S.) citizens aged 65 years or older; those eligible for disability; and/or subjects with end-stage renal disease. Excluded are beneficiaries who are also enrolled in a managed-care organization. Researchers often analyze Medicare SAF data because it provides a comprehensive snapshot of healthcare use and expenditures among the elderly in the U.S.

The SAFs are constructed from weekly data submissions to the National Claims History 100% Nearline File. The SAF database includes medical claims submitted for laboratory tests, inpatient hospital stays, outpatient care, physician care, skilled nursing facility care, home health care, durable medical equipment use, hospice care, and a denominator file that includes beneficiaries' demographic characteristics (age, sex, race, etc.). Beneficiary IDs are encrypted in this file and remain consistent between all claims in a given year and across years. We studied years 2008 through 2011 in Medicare FFS Carrier (Part B), Inpatient, Outpatient and Denominator files.

Patients were included in the evaluation if the beneficiary had an index Holter in 2009 and was enrolled in Medicare FFS Parts A and B in the 1 year prior to the date of the first Holter to date of death OR 2 years after the date of the index Holter event in 2009.

Exclusion criteria for a given beneficiary included the use of a Holter or other cardiac monitoring device one year prior to the purported date of the first Holter use event in 2009, diagnosis of AF 1 year prior to the date of the first Holter event in 2009, or a cardiac ablation procedure in the 1 year prior to the date of the first Holter event in 2009 (unless specified for supraventricular tachycardia (SVT), Wolff-Parkinson-White (WPW), or accessory bypass tract).

To illustrate how these criteria worked, consider the following example. A patient whose first Holter event occurred on February 1, 2009 would be retrospectively evaluated over the period February 1, 2008 through January 31, 2009 for exclusion criteria. The follow-up period for analyses would cover February 1, 2009 through February 1, 2011.

There were 46,840 beneficiaries with an initial Holter performed in 2009. After application of the exclusion criteria, the study cohort was reduced to 17,887 patients and the associated Consolidated Standards of Reporting Trials (CONSORT) diagram is shown in Figure 1.

Given the aims of the study, the following four areas of data collection were performed: diagnosis, additional monitoring, clinical events and costs. Diagnosis was identified by appropriate International Classification of Diseases, 9th Revision, Clinical Modification (ICD-9-CM) codes (Table 1) and additional ambulatory ECG monitoring by CPT codes and date of service. Clinical events were identified through site of service and ICD-9 codes for Emergency Department (ED), Inpatient and Observation (OBS) unit stays and ICD-9 codes for stroke, transient ischemic attack (TIA) and cardiac arrests. The principal diagnosis was used to determine clinical events. Costs were derived from the claims data in the Medicare SAFs.

The diagnostic sequence of events (which we characterized as the "Diagnostic Odyssey") was used to group patients into one of eight possible outcomes categories based on the occurrence of a clinical event, the ability of the Holter monitoring to provide a diagnosis, the use of repeat monitoring and the ultimate success in diagnosis and preventing clinical events. The tree of possibilities is given in Figure 2.

**Figure 1.** CONSORT Diagram

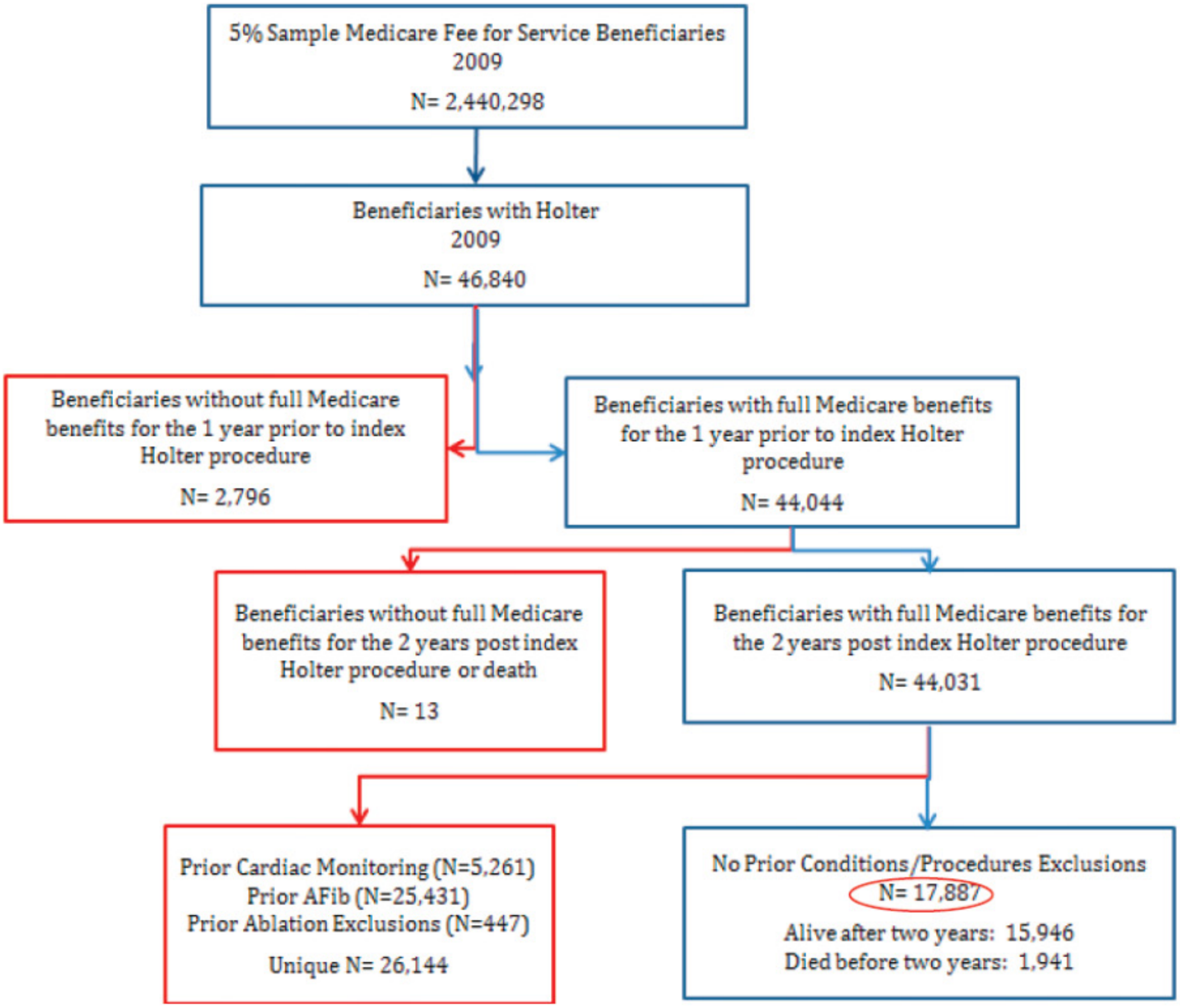

Afib: atrial fibrillation

**Table 1.** Arrhythmia Principal Diagnoses

| Diagnosis Code | Description | N | % (N=5,004)* |
|---|---|---|---|
| 427.31 | Atrial fibrillation | 1,466 | 29.3% |
| 427.89 | Other specified cardiac dysrhythmias | 1,300 | 26.0% |
| 427.9 | Cardiac dysrhythmia unspecified | 1,002 | 20.0% |
| 427.81 | Sinoatrial node dysfunction | 655 | 13.1% |
| 427.0 | Paroxysmal supraventricular tachycardia | 431 | 8.6% |
| 427.1 | Paroxysmal ventricular tachycardia | 238 | 4.8% |
| 427.32 | Atrial flutter | 102 | 2.0% |
| 427.42 | Ventricular flutter | 6 | 0.1% |

**Figure 2.** Outcomes Categories: Decision Tree – "Diagnostic Odyssey"

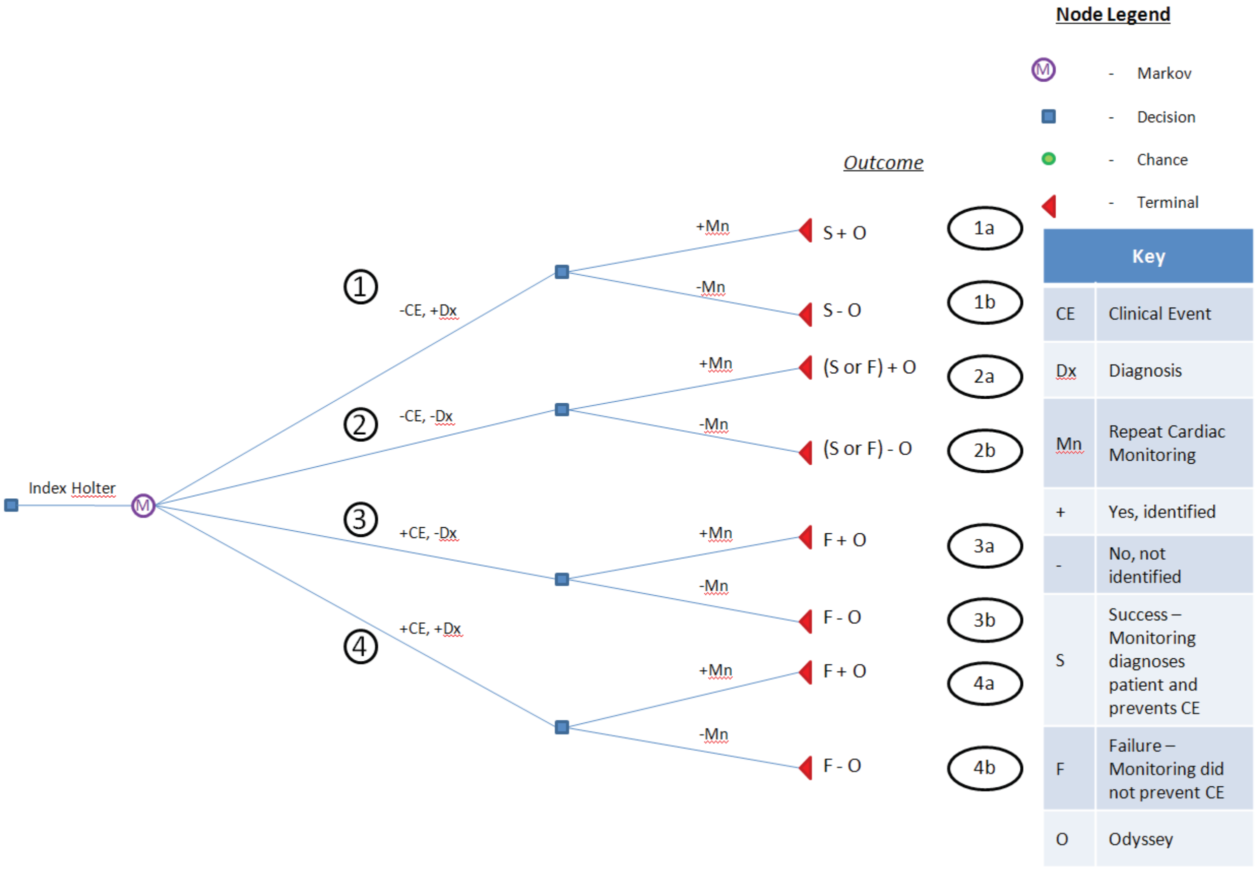

The 17,887 patients in the study sample were classified into each of these eight categories and the numbers and percentages tabulated. These were further sub-classified into survival through the study period (as described above). Finally the cost of care for the eight cohorts was determined from the Medicare SAF through the allowable charges in that database. From the combination of the percentage of patients in each category and their corresponding charges, it was possible to extrapolate to the general population of Holter monitor use. We also considered the frequency distributions of certain time to event outcomes, including time to first clinical event after the index Holter monitoring and the time to the first repeat Holter use after the index monitoring event. The former was categorized by the type of event, while the latter was classified by the monitor type. Our focus will be on the group of patients who, despite undergoing repeat cardiac monitoring, demonstrated no diagnoses or clinical events (Group 2a in Figure 2 and Table 2).

## RESULTS

Table 2 provides a summary of the patient counts and allowed charges for the entire study period for the eight categories ("Diagnostic Odyssey") of patients as defined in the Methods section.

The group of greatest interest is the "No results" category (Group 2a), since these 1,976 patients (11.1% of the total) reflected the failure of repeat Holter monitoring to either detect clinical events or diagnose disease. In spite of this failure, there was a total allowed charge of more than $45 million or slightly more than $23,000 per involved patient. When extrapolated over the entire Medicare FFS population with the given study criteria, this category was estimated to cost more than $900 million over the 2-year study period.

**Table 2.** "Diagnostic Odyssey" Patient Counts and Allowed Charges

| | | | Allowed Charges | | | | |
|---|---|---|---|---|---|---|---|
| | Branch | N Patients | Total | Average Per Patient | Total Inpatient | Total Outpatient | Total Part B |
| 2b | −CE, −Dx, −Mn | 8,074 | $115,244,713 | $4,274 | $29,210,291 | $28,792,599 | $57,241,823 |
| 3b | +CE, −Dx, −Mn | 2,385 | $63,144,546 | $26,476 | $22,073,938 | $14,748,296 | $26,322,311 |
| 2a | −CE, −Dx, +Mn | 1,976 | $45,987,782 | $23,273 | $14,150,566 | $10,424,886 | $21,412,330 |
| 1b | −CE, +Dx, −Mn | 1,924 | $48,291,936 | $25,100 | $19,400,990 | $10,684,998 | $18,205,949 |
| 4b | +CE, +Dx, −Mn | 1,459 | $65,653,071 | $44,999 | $34,017,088 | $11,397,619 | $20,238,364 |
| 1a | −CE, +Dx, +Mn | 1,156 | $30,187,408 | $26,114 | $11,344,771 | $6,323,153 | $12,519,484 |
| 4a | +CE, +Dx, +Mn | 465 | $19,170,535 | $41,227 | $7,810,190 | $4,055,323 | $7,305,021 |
| 3a | +CE, −Dx, +Mn | 448 | $14,620,310 | $32,635 | $5,418,757 | $3,696,332 | $5,505,220 |
| | Total | 17,887 | $402,300,299 | $22,491 | $143,426,591 | $90,123,207 | $168,750,501 |

Mn=repeat cardiac monitoring; CE=clinical event; Blue highlighted group (group 2a) is the "No odyssey" category.

Further examination of this group showed that Holter monitoring was the most commonly repeated test during the 2 years of follow-up (Figures 3 and 4). When a repeat test was performed, the large majority of the time it occurred in the first 3 months after the initial Holter test. Moreover, only one repeat test was typically performed.

**Figure 3.** Time to First Repeat Monitor Event after Index Holter by Monitor Type-Group 2a

MCOT: Mobile Cardiac Outpatient Telemetry; ILR: implantable loop recorder

**Figure 4.** Time to First Repeat Monitor Event after Index Holter by Monitor Type-Group 2a

MCOT: Mobile Cardiac Outpatient Telemetry; ILR: implantable loop recorder

Overall mortality in the sample was 10.9%. However, the mortality rate in the "No results" group, where no clinical event occurred in spite of the lack of diagnosis and additional monitoring, was lower—at about 7%.

## DISCUSSION

This study attempted to evaluate the clinical consequences and costs in Medicare patients who had no previous evidence of a cardiac arrhythmia and were undergoing their first Holter monitoring test. Using a very large database, the Medicare SAFs, we were able to identify a large cohort of 17,887 individuals who met the requirements of our defined population. The results of the study were stratified into several categories, which we will discuss individually below—the need for repeat monitoring, diagnosis attained versus not, and mortality.

### Repeat Monitoring

We found that about 23% of the total sample underwent repeat monitoring; most of this was of the Holter type and usually performed in a 3-month period after the first Holter procedure. Yet, in the Group 2a cohort, although there were no clinical events observed, there was also no diagnosis made. Thus, the value of repeat testing must be re-evaluated.

### No Diagnosis

As mentioned previously, the "No results" category (Group 2a), 11.1% of the total, reflected the failure of repeat Holter monitoring to either detect clinical events or diagnose disease. In spite of this failure, there was a total allowed charge of more than $45 million or slightly more than $23,000 per involved patient. When extrapolated over the entire Medicare FFS population with the given study criteria, this category was estimated to cost more than $900 million over the 2-year study period. This suggests that a large degree of effort, time and expense is spent on a relatively large group of individuals for which no obvious discernible clinical benefit is engendered.

**Mortality**

As noted previously, overall mortality in the sample was 10.9%, with one of the lowest values in Group 2a at 7.3%. Yet the use of initial or repeat monitoring did not have a diagnostic impact for this particular group. Again, the significance of repeat Holter monitoring was questionable insofar as mortality was concerned, and in spite of the more than $900 million outlay in this patient cohort for the two-year period in question.

In agreement with Kuhne[9], the diagnostic yield of Holter monitoring may not be as substantial as the prevailing clinical sentiment suggests. As a consequence, Healey and colleagues[4] have pointed out that subclinical outcomes such as tachyarrythmias have a significant association with the increased risk of AF along with stroke and systemic embolism. In the current study, we noted that repeat monitoring, often of the same type as done initially, frequently did not yield a diagnosis and patients continued to experience clinical events. Additional diagnostic paradigms need to be explored to improve these patient and system outcomes.

## ACKNOWLEGEMENT

We acknowledge the expert resources of Martin Lee, PhD in the preparation of this manuscript.

**Conflict of Interest Declaration**

Quorum Consulting, Inc., of which both Dr. Renée JG Arnold and Mr. Andrew Layton, are employees, received financial support for the writing of this manuscript from iRhythm Corporation.
